# Active learning for ontological event extraction incorporating named entity recognition and unknown word handling

**DOI:** 10.1186/s13326-016-0059-z

**Published:** 2016-04-27

**Authors:** Xu Han, Jung-jae Kim, Chee Keong Kwoh

**Affiliations:** School of Computer Engineering, Nanyang Technological University, 50 Nanyang Avenue, Singapore, 639798 Singapore; Data Analytics Department, Institute for Infocomm Research, 1 Fusionopolis Way, Singapore, 138632 Singapore

**Keywords:** Active learning, Biomedical natural language processing, Information extraction

## Abstract

**Background:**

Biomedical text mining may target various kinds of valuable information embedded in the literature, but a critical obstacle to the extension of the mining targets is the cost of manual construction of labeled data, which are required for state-of-the-art supervised learning systems. Active learning is to choose the most informative documents for the supervised learning in order to reduce the amount of required manual annotations. Previous works of active learning, however, focused on the tasks of entity recognition and protein-protein interactions, but not on event extraction tasks for multiple event types. They also did not consider the evidence of event participants, which might be a clue for the presence of events in unlabeled documents. Moreover, the confidence scores of events produced by event extraction systems are not reliable for ranking documents in terms of informativity for supervised learning. We here propose a novel committee-based active learning method that supports multi-event extraction tasks and employs a new statistical method for informativity estimation instead of using the confidence scores from event extraction systems.

**Methods:**

Our method is based on a committee of two systems as follows: We first employ an event extraction system to filter potential false negatives among unlabeled documents, from which the system does not extract any event. We then develop a statistical method to rank the potential false negatives of unlabeled documents 1) by using a language model that measures the probabilities of the expression of multiple events in documents and 2) by using a named entity recognition system that locates the named entities that can be event arguments (e.g. proteins). The proposed method further deals with unknown words in test data by using word similarity measures. We also apply our active learning method for the task of named entity recognition.

**Results and conclusion:**

We evaluate the proposed method against the BioNLP Shared Tasks datasets, and show that our method can achieve better performance than such previous methods as entropy and Gibbs error based methods and a conventional committee-based method. We also show that the incorporation of named entity recognition into the active learning for event extraction and the unknown word handling further improve the active learning method. In addition, the adaptation of the active learning method into named entity recognition tasks also improves the document selection for manual annotation of named entities.

## Background

A common framework of information extraction systems is supervised learning, which requires training data that are annotated with information to be extracted. Such training data are usually manually annotated, where the annotation process is time-consuming and expensive. On the other hand, in biomedical domain, recent research efforts on information extraction are extending from focusing on a single event type such as protein-protein interaction (PPI) [1] and gene regulation [2] to simultaneously targeting more complicated, multiple biological events defined in ontologies [3], which makes the manual annotation more difficult. There is thus the need of reducing the amount of annotated data that are required for training event extraction systems.

Active learning is the research topic of choosing ‘informative’ documents for manual annotation such that the would-be annotations on the documents may promote the training of supervised learning systems more effectively than the other documents [4]. It has been studied in many natural language processing applications, such as word sense disambiguation [5], named entity recognition [6–8], speech summarization [9] and sentiment classification. Its existing works can be roughly classified into two approaches: uncertainty-based approach [10] and committee-based approach [11]. The uncertainty-based approach is to label the most uncertain samples by using an uncertainty scheme such as entropy [12]. It has been shown, however, that the uncertainty-based approach may have worse performance than random selection [13–15].

In the biomedical information extraction, the uncertainty-based approach of active learning has been applied to the task of extracting PPIs. For instance, [16] proposed an uncertainty sampling-based approach of active learning, and [17] proposed maximum uncertainty based and density based sample selection strategies. While the extraction of PPI is concerned with a single event type of PPI, however, recent biomedical event extraction tasks [18] involve multiple event types, even hundreds of event types in the case of the Gene Regulation Ontology (GRO) task of BioNLP-ST’13 [19].

The committee-based approach, based on a committee of classifiers, selects the documents whose classifications have the greatest disagreements among the classifiers and passes them to human experts for annotation. This approach, however, has several issues in adaptation for event extraction tasks. First, event extraction (e.g. PPI extraction, gene regulation identification) is different from many other applications of active learning, which are in essence document classification tasks. Event extraction is to locate not only event keywords (e.g. bind, regulates), but also event participants (e.g. gene/protein, disease) within documents and to identify pre-defined relations between them (e.g. subject-verb-object). Thus, even if the event extraction systems produce confidence scores for its resultant events, the confidence scores do not correspond to the probability of how likely a document expresses an event type: in other words, how likely a document belongs to an event type class, which should be the goal of classifiers of the committee-based approach for event extraction. Second, previous classifiers for the committee-based approach may miss some details of events including event participants. For example, the keyword ‘expression’ may mislead a classifier to predict that the document with the keyword expresses gene expression event, although the document does not contain any gene name.

Our target tasks of event extraction for active learning in this paper are those introduced in BioNLP-ST’13 [20], which involve multiple, complicated event types. Currently, there is only one event extraction system available for all the tasks, called TEES [21], and we need an additional classifier to follow the committee-based approach.

We thus propose as an additional classifier a novel statistical method for informativity estimation, which predicts how likely a text expresses any event concept of a given ontology. The method is based on a language model for co-occurrences between n-grams and event concepts. Furthermore, it independently estimates the presence of event participants in a text and the probabilities of out-of-vocabulary words and combines them with the prediction of event concept in the text. We collectively estimate the informativity of a text for all the concepts in a given ontology, similarly to the uncertainty-based approach of [22–24].

We also present a revised committee-based approach of active learning for event extraction, which combines the statistical method with the TEES system as follows: Since the confidence scores of the TEES system are not reliable for active learning, we take TEES outputs as binary, that is, whether the system extracts any instance of a concept from a text or not. The disagreement between the TEES system and the statistical model is captured when, given a text (T) and an event concept (C), the TEES system does not extract any instance of C in T, but the probabilistic model predicts a high probability of C in T. In other words, the TEES system is used for filtering potential false positives, and the probabilistic model for ranking them.

We further adapt our active learning method and the statistical method for event concept detection to named entity recognition, including gene name recognition. We show that our method can improve active learning for named entity recognition as well, when tested against the BioCreative and CoNLL datasets.

## Methods

We formalize the general workflow of active learning as follows: At the start of round *t*, let $\mathcal {U}^{(t-1)}$ be the pool of unlabeled documents and let $\mathcal {L}^{(t-1)}$ be the pool of labeled documents, where *t* starts from 1. In round *t*, we select the most ‘informative’ document *x*^(*t*)^ from $\mathcal {U}$, manually label it, and add it to $\mathcal {L}$. If the label *y*^(*t*)^ is assigned to the document *x*^(*t*)^ by the oracle, the labeled and unlabeled document sets are updated as follows:

(1) $$  \mathcal{L}^{(t)} = \mathcal{L}^{(t-1)} \cup \{(x^{(t)}, y^{(t)})\}\qquad\qquad \mathcal{U}^{(t)} = \mathcal{U}^{(t-1)} \backslash x^{(t)}  $$

Such process is iterated until a certain stopping criteria is met, such as when $\mathcal {U} = \emptyset $ and after a pre-defined number of rounds. It also can be done in a batch mode, where a group of documents are selected at each round for the manual labeling.

### Active learning method for event extraction

**Fig. 1:**
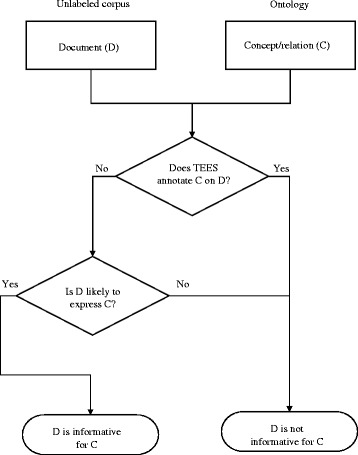
Overview of proposed active learning method. The integration of underlying system into active learning method. For event extraction task, if the underlying event extraction system (TEES) can recognize the concept (C) in the given document (D), the D is not considered as informative

As explained above, our active learning method follows the committee-based approach. As the committee, we employ two classifiers: A classifier based on an event extraction system called TEES and a statistical classifier based on language modeling (see the next section for details). The TEES [21] is a state-of-the-art biomedical event extraction system based on support vector machine, and was the only system that participated in all the tasks of BioNLP-ST’13, showing the best performance in many of the tasks [25]. The TEES system produces the confidence score of each event it extracts. However, we do not use the score for active learning because the confidence score does not indicate the probability of the event in the document. We also assume that if the TEES system extracts an event (E) from a document (D), D is not informative for E, because true positives are already not informative and because the correction (i.e. labeling) of false positives might not be useful for training event extraction systems where event descriptions are scarce, and thus there are far more negative examples than positive examples. In other words, the primary goal of our active learning method is to correct more false negatives, that is, to annotate the true events not extracted by the existing system. Figure 1 depicts the workflow of the proposed method.

Our method works iteratively as follows: In round *t*, we train the TEES system and the statistical classifier based on $\mathcal {L}^{(t-1)}$. We measure the informativity of each unlabeled document among $\mathcal {U}^{(t-1)}$ and choose the top documents as feed for manual annotation. We measure the informativity score of a document at the sentence level, that is, the average of the informativity scores of all the sentences in the document, as illustrated in (2).

(2) $$  {}x_{Informativity}^{*}=\underset{x}{argmax} ~I_{\theta^{t}}(x) \qquad\quad I_{\theta^{t}}(x) = \frac{1}{||x||} \sum\limits^{S_{k} \in x}I(S_{k})  $$

*θ*^*t*^ indicates the current models of the TEES system and the statistical classifier at round *t*, but we will omit it for simplicity.

The informativity of a sentence (*S*_*k*_) is measured for the event concept set $\mathcal {E}$, which contains all event defined in a given ontology, as expressed in (3). The informativity score for event concept set is denoted as $I(S_{k},\mathcal {E})$. In fact, the BioNLP-ST’13 tasks include not only events, but also relations. A key difference between events and relations is that an event always involves an event keyword (e.g. ‘regulates’ for GeneRegulation), but a relation does not have any keyword (e.g. partOf). For simplicity, we mention only events in the paper, while our method involves both events and relations in the same way.

(3) $$ I(S_{k}) = I(S_{k},\mathcal{E})  $$

#### Informativity for event concept set

The informativity of a sentence for event concept set is calculated as the sum of the informativity scores of the sentence for all the event as follows:

(4) $$ I(S_{k},\mathcal{E}) = \sum\limits^{E_{i} \in \mathcal{E}} I(S_{k},E_{i})  $$

As explained earlier, we treat a sentence as non-informative for an event if the event extraction system TEES can extract any instance of the event from the sentence. Otherwise, the informativity score is estimated as the probability of the concept given the sentence as follows:

(5) $$ \begin{aligned}  {\kern-16.5pt}I(S_{k},E_{i}\!) \,=\,\! \left\{\!\! \begin{array}{ll} 0 \quad & \text{if}\,\, E_{i}\, \text{is recognized in}\,\, S_{k}\,\, \text{by the TEES model at round}\,\, \textit{t}\\ p(E_{i}|S_{k}) \quad & otherwise \end{array} \right. \end{aligned}  $$

*p*(*E*_*i*_|*S*_*k*_) can be converted into (6) using the Bayes’ theorem.

(6) $$  p(E_{i}|S_{k}) = \frac {p(E_{i})p(S_{k}|E_{i})}{p(S_{k})}  $$

The *P*(*E*_*i*_) is estimated using the maximum-likelihood estimation (MLE) based on the statistics of event annotations in the training data.

As for *P*(*S*_*k*_|*E*_*i*_), we score the correlation between the sentence *S*_*k*_ and the event *E*_*i*_ with a real value scoring function *Z* (see below for details) and use the softmax function to represent it as a probabilistic value, shown in (7).

(7) $$  \begin{aligned} {}p(S_{k}|E_{i}) &=\sigma(Z(S_{k}:E_{i}))=\frac{1}{\sum_{E_{j} \in \mathcal{E}} \exp(Z(S_{k}:E_{j}))} \exp(Z(S_{k}:E_{i})) \end{aligned}  $$

We use two types of units to approximately represent the sentence *S*_*k*_: n-grams (NG) and predicate-argument relations (PAS) produced by the Enju parser [26]. A sentence is represented as a bag of elements of a unit, for example, a bag of all n-grams or a bag of all predicate-argument relations from the sentence.

##### A. Using N-gram feature for probability estimation

If we use the bag of n-gram model, the score *Z*(*S*_*k*_:*E*_*i*_) is measured using the average of the correlation score between the n-gram (NG) contained in the sentence with the event, expressed in (8), where len(*S*_*k*_) is the normalization factor and is calculated as the word count of sentence *S*_*k*_.

(8) $$ Z(S_{k}:E_{i})= \frac{1}{len(S_{k})} \sum\limits^{NG_{j} \in S_{k}} p(NG_{j}|E_{i})  $$

While the probability between the n-gram and event *p*(*NG*_*j*_|*E*_*i*_) is also calculated using a correlation score *W*(*NG*_*j*_,*E*_*i*_) between the n-gram and the event, together with the softmax function, shown in (9).

(9) $$  \begin{aligned} {}p(NG_{j}|E_{i}) &=\sigma(W(NG_{j},E_{i}))\\ &= \frac{1}{\sum_{NG_{l} \in \mathcal{U}} \exp(W(NG_{l},E_{i}))} \exp(W(NG_{j},E_{i})) \end{aligned}  $$

The correlation score *W*(*NG*_*j*_,*E*_*i*_) is calculated using one of the following three methods: 1) Yates’ chi-square test, 2) relative risk, and 3) odds ratio [27]. For the calculation of the three methods, a 2 ×2 table is constructed for each pair of an N-gram and an event at the level of sentences, as shown in Table 1. For example, *a* indicates the number of sentences that contain the N-gram *NG*_*j*_ and express the event *E*_*i*_.

**Table 1:** Numbers of sentences for the calculation of correlation score between *E*_*i*_ and *N* *G*_*j*_

	Express event *E*_*i*_	Not express event *E*_*i*_	Total
Contain N-gram *N* *G*_*j*_	a	b	*N*_*A*_
Not contain N-gram *N* *G*_*j*_	c	d	*N*_*B*_
Total	*N*_*S*_	*N*_*F*_	N

Based on the 2 ×2 table, the three methods of Yates’ chi-square test, relative risk, and odds ratio calculate the correlation score for the pair as shown in the formulas (10), (11), and (12), respectively.

(10) $$ W(NG_{j},E_{i}) = \frac{N (|ad - bc| - N/2)^{2} }{N_{S} N_{F} N_{A} N_{B}}  $$

(11) $$ W(NG_{j},E_{i}) = \frac{a / (a + b) }{ c / (c + d)}  $$

(12) $$ W(NG_{j},E_{i}) = \frac{a / b }{ c / d}  $$

##### B. Using predicate-argument relation for probability estimation

Similarly for the bag of predicate-argument relation model, the score *Z*(*S*_*k*_:*E*_*i*_) is calculated with the average of the correlation scores between the event and the predicate-argument relations from the sentence, as in (13).

(13) $$ Z(S_{k}:E_{i})= \frac{1}{len(S_{k})} \sum\limits^{PAS_{j} \in S_{k}} p(PAS_{j}|E_{i})  $$

### Additional features of active learning

We introduce two additional features of our active learning method: Incorporation of event participants and dealing with out-of-vocabulary words.

#### Incorporation of event participants

The absence of event participants should negatively affect the prediction of events. To reflect this observation, we utilized a gene name recognition system, called Gimli [28], in order to recognize gene/protein names, since most of the BioNLP shared tasks involve genes and proteins (e.g. gene expression, gene regulation, phosphorylation). We incorporate the results of the Gimli system into our active learning method as follows:

(14) $$ I(S_{k}) = I(S_{k}, \mathcal{E}, NG) + I(S_{k}, \mathcal{N})  $$

(15) $$ I(S_{k}, \mathcal{N}) = \delta \times T  $$

*T* indicates the number of gene/protein names predicted in a sentence *S*_*k*_.

In fact, the Gimli system can be replaced with other named entity recognition systems for tasks whose event participants are other than gene/protein. Since the event extraction tasks for evaluating our active learning method (i.e. BioNLP shared tasks) are mainly about gene/protein, we do not replace the Gimli system when evaluating the incorporation of event participants. When we apply our active learning method for the tasks of named entity recognition (NER), however, we will evaluate it against two NER systems (i.e. Gimli, Stanford NER system) (see for details Sections ‘Active learning method for NER task’ in Page 8, ‘Datasets and employed systems’ in Page 11, and ‘Evaluation of active learning method for NER task’ in Page 19).

#### Dealing with OOV issue with word similarity

When we use the n-gram features, there is Out-of-Vocabulary (OOV) issue, such that some n-grams in the test dataset may not appear in the training dataset. To tackle this issue, we adopt the word2vec system, which is an unsupervised method for representing each word as a vector in a latent semantic model and for measuring word similarity [29], as follows: Consider an n-gram *NG*_*out*_ that does not occur in the training dataset. We use word2vec to find the top-k n-grams *NG*_*in*_ that are closest to *NG*_*out*_, where the word similarity score between *NG*_*out*_ and each *NG*_*in*_ is designated as *Sim*(*NG*_*out*_,*NG*_*in*_). We then recalculate the correlation scoring function *W*(*NG*_*out*_,*E*_*i*_) as shown in Formula (16). Note that since word2vec can only handle unigrams, and also since unigrams show the best performance in our experiments of parameter optimization (see the next section), we only deal with unknown unigrams in this method. The word similarity scores are trained a priori using the whole set of MEDLINE abstracts released in April 2014.

(16) $$ \begin{aligned} {}W_{OOV}(NG_{out},E_{i}) \!= \sum\limits^{NG_{in} \in Training Dataset}_{top-k} W\!(NG_{in},E_{i})\! \times Sim_{NG_{out}, NG_{in}} \end{aligned}  $$

We denote the n-gram-based informativity of sentence calculated using the updated correlation scoring function (16) as *I*(*S*_*k*_,*NG*_*OOV*_). For example, when the correlation scoring function in (9) is updated, the resultant informativity in (4) is denoted as $I(S_{k}, \mathcal {E}, NG_{OOV})$.

#### Linear combination of n-gram and predicate-structure relation features

While we choose either n-grams or predicate-argument relations as features, we also tested the linear combination of the two feature sets for our active learning method, as follows:

(17) $$  \begin{aligned} {}I(S_{k}) &= \alpha \times I (S_{k}, NG_{OOV}) + \beta \times I(S_{k}, PAS) + \gamma \times I(S_{k}, \mathcal{N})\\ &= \alpha \times I(S_{k}, \mathcal{E}, NG_{OOV}) + \beta \times I(S_{k}, \mathcal{E}, PAS) + \gamma \times I(S_{k}, \mathcal{N}) \end{aligned}  $$

Table 2 illustrates the calculation of informativity scores in pseudo codes.

**Table 2:** Proposed algorithm of active learning with TEES

Input: labeled document pool *L*, unlabeled document pool *U*, batch size *b*
**// Initialization**
*E* *R*_0_ = the set of events/relations annotated on *L*
Learn a TEES model *M*_0_ from *E* *R*_0_
*i* = 0 // the index of the current round
**// Active Learning Loop**
**while** *U* is **not** empty:
*i* += 1
**for** each document *D*_*ij*_ in *U*:
Document informativity score *I*(*D*_*ij*_)=0
**for** each sentence *S*_*k*_ in *D*_*ij*_:
Apply *M*_*i*−1_ to *S*_*k*_ and collect the resultant events/relations set $ER_{S_{k}}$
**for** each event/relation *er* s.t. *er* $\notin ER_{s_k}$:
*I*(*D*_*ij*_) += informativity score *I*(*S*_*k*_,*e* *r*)
*I*(*D*_*ij*_) = *I*(*D*_*ij*_) / sizeOf(*D*_*ij*_)
Rank *D*_*ij*_ in *U* based on *I*(*D*_*ij*_) and select the top *b* documents,
designated as *B*
Remove *B* from *U*, add *B* to *L*, and add the annotations on *B* to *E* *R*_*i*−1_,
designated as *E* *R*_*i*_
Learn a new model *M*_*i*_ from *E* *R*_*i*_

### Active learning method for NER task

We also adapt our active learning method to named entity recognition (NER), considering the ontology concepts of named entities (e.g. Gene, Disease) instead of events (e.g. PPI, gene regulation). The method for named entity recognition estimates informativity, or the likelihood of a text expressing any named entities.

Similar to Eq. (2), the informativity estimation in the NER task is expressed in (18).

(18) $$ {}x_{Infomativity}^{*}=\underset{x}{argmax} ~I_{\theta^{t}}(x)\qquad\quad I_{\theta^{t}}(x) = \frac{1}{||x||} \sum\limits^{S_{k} \in x}I(S_{k})  $$

*θ*^*t*^ indicates the current model of a given NER system and the statistical classifier at round *t*, but we will omit it for simplicity. We evaluate our method with two NER systems of Gimli for biomedical domain and Stanford NER system for general domain (see Section “Results and discussion” for details of evaluation), one system at a time.

The informativity of a sentence for named entity set is calculated as the sum of the informativity scores of the sentence for all the named entities as follows:

(19) $$ I(S_{k}) = I(S_{k},\mathcal{N}) = \sum\limits^{N_{i} \in \mathcal{N}} I(S_{k},N_{i})  $$

Similar to the active learning method for event extraction, we treat a sentence as non-informative for an named entity if the NER system can recognize any instance of the named entity from the sentence. Otherwise, the informativity score is estimated as the probability of the named entity given the sentence as follows:

(20) $$ \begin{aligned}  {}I\!(S_{k},N_{i}) \,=\,\! \left\{\! \begin{array}{ll} 0 \quad & \text{if}\, N_{i}\, \text{is recognized in}\, S_{k}\, \text{by the NER system at round} \,\textit{t}\\ p(N_{i}|S_{k}\!) \quad & otherwise \end{array} \right. \end{aligned}  $$

The probability *p*(*N*_*i*_|*S*_*k*_) is calculated as follows:

(21) $$ p(N_{i}|S_{k}) = \frac{p(N_{i})p(S_{k}|N_{i})}{p(S_{k})}  $$

Similarly to the estimation for event, *p*(*N*_*i*_) is estimated using the maximum-likelihood estimation (MLE) based on the statistics of named entities in the training data. For the calculation of *p*(*S*_*k*_|*N*_*i*_), we follow similar steps as in (7), using n-grams (i.e. Formula (8)), but not using PAS (i.e. Formula (13)).

### Comparison with related works

In this section, we describe the previous methods of active learning that we compare with our proposed methods for event extraction in the evaluation experiments.

**A. Conventional committee-based method**

The committee based active learning, based on a committee of classifiers, selects the documents whose classifications have the greatest disagreements among the classifiers and passes them to human experts for annotation, expressed as follows:

(22) $$ x_{Committee}^{*}=\underset{x}{argmax} ~D_{\theta}(Y|x)  $$

*D*_*θ*_(*Y*|*x*) is the disagreements among the classifiers for a document *x* under the model *θ*, and the *Y* is the whole label set. We use the summation of disagreement over the sentence *S*_*k*_ contained in the document *x*.

(23) $$ D_{\theta}(Y|x) = \sum\limits^{S_{k} \in x} D(Y|S_{k})  $$

For each sentence, we measure the collective disagreement over the whole event concept set $\mathcal {E}$ defined in the ontology by using the sum of all disagreement for all event *E*_*i*_.

(24) $$ D(Y|S_{k}) = \sum\limits^{E_{i} \in \mathcal{E}} D(E_{i}|S_{k})  $$

The disagreement *D*(*E*_*i*_|*S*_*k*_) is calculated using the absolute value of the differences of the probability produced by the classifiers, named the aforementioned informativity estimation method and the TEES event extraction system.

(25) $$ D(E_{i}|S_{k}) = | p_{Informativity}(E_{i}|S_{k}) - p_{TEES}(E_{i}|S_{k}) |  $$

The *p*_*TEES*_(*E*_*i*_|*S*_*k*_) is the probability estimated from the TEES system, and *p*_*Informativity*_(*E*_*i*_|*S*_*k*_) is from the informativity estimation using statistical method, which is calculated in Eq. (6). Note that while *p*(*E*_*i*_|*S*_*k*_) in Eq. (5) is estimated using Eq. (6) only for the sentences from which no *E*_*i*_ is recognized by the TEES, the same informativity probability in Eq. (25) is estimated for all the sentences of unlabeled documents.

However, as the TEES is a support vector machine (SVM) based system and do not produce probabilistic output, we use the confidence the SVM classifier has in its decision for a event prediction as follows:

(26) $$ \begin{aligned} {}p_{TEES}(E_{i}|S_{k}) =\!\sigma(C(E_{i}|S_{k}))=\frac{1}{\sum_{E_{j} \in \mathcal{E}} \exp(C(E_{j}|S_{k}))} \exp(C(E_{i}|S_{k})) \end{aligned}  $$

*C*(*E*_*i*_|*S*_*k*_) is the confidence for the classifier.

The confidence is calculated using the **difference-2** of the distance from the separating hyperplane, produced by the SVM classifier. It is shown to have best performance in active learning [30, 31], and the calculation is expressed as follows:

(27) $$  \begin{aligned} &m_{max} = \underset{m}{argmax} ~dist(m,S_{k}) \\ &n = \underset{n \neq m_{max}}{argmax} ~dist(n,S_{k}) \\ &C(E_{i}|S_{k}) = dist(m_{max},S_{k}) - dist(n,S_{k}) \\ \end{aligned}  $$

The *dist*(*m,S*_*k*_) is the distance of the predicted label *m* in such sentence *S*_*k*_.

Similarly in adapting to the NER task, for each sentence, we measure the collective disagreement over the whole named entity concept set $\mathcal {N}$ by using the sum of all disagreement for all named entity *N*_*i*_.

(28) $$ D(Y|S_{k}) = \sum\limits^{N_{i} \in \mathcal{N}} D(N_{i}|S_{k})  $$

The disagreement *D*(*N*_*i*_|*S*_*k*_) is calculated using the absolute value of the differences of the probability produced by the classifiers, named the aforementioned informativity estimation method and the NER system.

(29) $$  D(N_{i}|S_{k}) = | p_{Informativity}(N_{i}|S_{k}) - p_{NER}(N_{i}|S_{k}) |  $$

The *p*_*NER*_(*N*_*i*_|*S*_*k*_) is the marginal probability provided by the Conditional Random Field (CRF) model from the NER system, and *p*_*Informativity*_(*N*_*i*_|*S*_*k*_) is from the informativity estimation using statistical method.

**B. Entropy based active learning method**

Entropy is the most common measure for uncertainty, which indicates a variable’s average information content. The document selection of entropy-based methods is formalized as follows:

(30) $$ x_{Entropy}^{*} =\underset{x}{argmax} ~H_{\theta}(Y|x)  $$

The *H*_*θ*_(*Y*|*x*) is the entropy of a document *x* under the model *θ* and the *Y* is the whole label set. We use the summation of entropy over the sentence *S*_*k*_ contained in the document *x*.

(31) $$ H_{\theta}(Y|x) = \sum\limits^{S_{k} \in x} H(Y|S_{k})  $$

For each sentence *S*_*k*_, we use the aforementioned bag of n-gram method, and estimate *H*(*Y*|*S*_*k*_) as the average entropy of each n-gram *NG*_*j*_ in *S*_*k*_, as follows:

(32) $$  H(Y|S_{k}) = \frac{1}{len(S_{k})} \sum\limits^{NG_{j} \in S_{k}} H(Y|NG_{j})  $$

We estimate the collective entropy over the whole event concept set $\mathcal {E}$ defined in the ontology as the summation of the entropy for all event *E*_*i*_.

(33) $$  H(Y|NG_{j}) = \sum\limits^{E_{i} \in \mathcal{E}} H(E_{i}|NG_{j})  $$

*H*(*E*_*i*_|*NG*_*j*_) is calculated by using the Weka package for the calculation of entropy [32].

**C. Gibbs error based active learning method**

Gibbs error criterion is shown to be effective for active learning [33], which selects documents that maximize the Gibbs error, as follows:

(34) $$ x_{Gibbs}^{*} =\underset{x}{argmax} ~ H_{Gibbs}(\theta)  $$

Similarly to the entropy-based method implementation, we calculate the collective Gibbs error as follows:

(35) $$ \begin{aligned} {}H_{Gibbs}(\theta)\! =\!\! \sum\limits^{S_{k} \in x} \!H_{Gibbs}(Y|S_{k}) \,=\,\! \sum\limits^{S_{k} \in x}\!\! \frac{1}{len(S_{k})}\!\! \sum\limits^{NG_{j} \in S_{k}} \sum\limits^{E_{i} \in \mathcal{E}} \!H_{Gibbs}(E_{i}|NG_{j}) \end{aligned}  $$

For the calculation of *H*_*Gibbs*_(*E*_*i*_|*NG*_*j*_), we use the conditional probability of *p*(*E*_*i*_|*NG*_*j*_), defined as follows [33], where *p*(*E*_*i*_|*NG*_*j*_) is estimated using the proposed method as shown in (9):

(36) $$ H_{Gibbs}(E_{i}|NG_{j}) = 1 - {p(E_{i}|NG_{j})}^{2}  $$

## Results and discussion

### Datasets and employed systems

The BioNLP shared tasks (BioNLP-ST) were organized to track the progress of information extraction in the biomedical text mining. In this paper, we used the datasets of three tasks, namely GRO’13 (Gene Regulation Ontology) [19], CG’13 (Cancer Genetics) [34] and GE’13 (Genia Event Extraction) [35]. Each corpus was manually annotated with an underlying ontology, whose number of concepts and hierarchy are different from each other. A comparison between the datasets is given in Table 3. Note that since the official test datasets for CG and GE tasks are inaccessible, we instead use parts of their training datasets as the ‘test’ datasets, and the statistics of the datasets include only those accessible documents.

**Table 3:** Summary of task datasets used in the experiments

Task	Corpus size (Dev/Train/Test)	Document type	No. event concepts	No. relations
GRO’13	300 (50/150/100)	MEDLINE abstract	507	10
CG’13	400 (100/200/100)	MEDLINE abstract	58	1
GE’13	20 (5/10/5)	PubMed Central full text	13	20

Specifically, we employ the state-of-the-art Stanford NER [36] system for the CoNLL-2003 [37] dataset, and the Gimli gene name recognition system [28] for the BioCreative II Gene Mention [38] dataset. Note that in BioCreative task, the named entities are naturally of one class, i.e., the Gene/Protein name; while the CoNLL dataset involves four classes of named entities (i.e. Person, Organization, Location, Misc).

### Evaluation metrics for comparison of active learning methods

To compare the performance of the different strategies of sample selection, we plot their performance in each iteration. Since the difference between some plots is not obvious, however, we mainly use the evaluation metric of *deficiency* for comparison [39, 40], defined as follows:

(37) $$ Def_{n}(AL,REF)=\frac{\sum_{t=1}^{n}(acc_{n}(REF)-acc_{t}(AL))}{\sum_{t=1}^{n}(acc_{n}(REF)-acc_{t}(REF))}  $$

The *acc*_*t*_(*C*) is the performance of the underlying classifier *C* at *t*^*th*^ round of learning iteration. AL is an active learning method, and REF is a baseline method (see below for details). *n* refers to the total number of rounds (i.e. 10). A deficiency value smaller than 1.0 means that the active learning method is superior to the baseline method, and in general, a smaller value indicates a better method.

### Parameter optimization

We first take a parameter optimization step to determine the most appropriate parameters for the aforementioned calculation of informativity scores.

#### Correlation measure and n-gram size

As mentioned above, we considered three correlation measures to estimate the correlation score between n-gram and event, including chi-square test, relative risk, and odds ratio. We also should determine the value of *n* for *n*-grams. To find the optimal solutions for the two tasks, we carried out a simulation of ontology concept prediction at the sentence level as follows: Given a sentence *S*_*i*_ and *N*_*i*_ ontology concepts manually annotated on the sentence, we predict the top *N*_*i*_ ontology concepts in *S*_*i*_ and compare them with the *N*_*i*_ manually annotated concepts, measuring the overlap between the two concepts sets. We select the best combination of co-occurrence analysis method and n-gram size for the rest of experiments in this paper.

Using 10-fold cross validation, the average prediction rate is calculated and reported in Table 4. Each column corresponds to an n-gram size, and each row to one of the three co-occurrence analysis methods used for the prediction. Note that when N=2 (i.e. bi-grams), it does not include unigrams for the calculation. N=1-2 indicates the mixture of unigrams and bi-grams. This experiment is carried out using the GRO’13 dataset.

**Table 4:** Parameter optimization results

Calculation method	N-gram					
	*N*=1	*N*=2	*N*=3	*N*=4	*N*=5	*N*=1−2
Chi-square	**0.507**	0.413	0.159	0.036	0.009	0.436
Relative ratio	0.341	0.395	0.307	0.128	0.038	0.361
Odds	0.420	0.395	0.274	0.117	0.035	0.407

The averaged concept prediction accuracy is reported. The best accuracy is highlighted in boldface

As shown in Table 4, for all co-occurrence analysis methods, the accuracy mostly drops as the length of N-grams increases. This may happen due to the data sparseness problem for large N-grams. We choose to use **chi-square test** and **unigrams** for the following experiments based on the results.

#### Parameter for the incorporation of event participants

The parameter of *δ* in Eq. (15) is to determine the significance of effects of event participants on event concept prediction. We tested our active learning method in Eq. (14) against the GRO’13 dataset with the *δ* values set as 0.15, 0.25 and 0.35. We summarize the performance results in terms of deficiency in Table 5. We choose the *δ*=0.25 for the following experiments based on the results.

**Table 5:** Parameter optimization results

Method	GRO’13
RS_Average	1
*δ* = 0.15	0.716
*δ*= 0.25	**0.706**
*δ* = 0.35	0.713

The deficiencies of active learning method using different factor against the GRO’13 are reported. The best deficiency is highlighted in boldface in this table and also in the tables below

#### Parameter for dealing with OOV issue

In dealing with the OOV issue, we choose top-*k* similar words for an unknown word, as in Formula (16). In order to choose the optimal value for *k*, we use the linear combination method in Eq. (17) with the other parameters *α*=0.1,*β*=0.1 and *γ*=0.8, and test our active learning method against the GRO’13 dataset, as changing the *k* value from 5 to 25. We summarize the deficiency of the active learning method using the different *k* values in Table 6. As the result, we choose *k*=25 for the remaining experiments.

**Table 6:** Parameter optimization results

Method	GRO’13
RS_Average	1
LC_(*α*=0.1, *β*=0.1, *γ*=0.8), *k* = 5	0.611
LC_(*α*=0.1, *β*=0.1, *γ*=0.8),*k* = 10	0.600
LC_(*α*=0.1, *β*=0.1, *γ*=0.8),*k* = 15	0.617
LC_(*α*=0.1, *β*=0.1, *γ*=0.8),*k* = 20	0.628
LC_(*α*=0.1, *β*=0.1, *γ*=0.8),*k* = 25	**0.563**

The deficiencies of active learning method using different factor against the GRO’13

### Evaluation of active learning methods for event extraction

#### Active learning methods using informativity estimation

In the following evaluations, we show the learning curves and deficiencies of the event extraction system TEES under different sample selection strategies against the dataset of GRO’13, CG’13 and GE’13 task. The active learning methods use only the informativity estimation, but not the additional features such as incorporation of event participants and dealing with OOV issue, which will be discussed in the next section.

We compare the proposed active learning method with other sample selection strategies, including random selection, and entropy-based [17], and Gibbs error [33] based, as well as a conventional committee based active learning methods. We use the random selection as the baseline for deficiency calculation. Each experiment has ten rounds, where in each round, 10 % of the original training data are added for training the TEES system. The initial model of the TEES system before the first round is trained only on the development dataset. Note that the test data of each dataset is fixed. The followings are considered for the selection of additional 10 % training data in each round:

- Random selection: We randomly split the training data into 10 bins in advance, and during the training phase in each round, one bin is randomly chosen. We report the averaged performance of random selection for ten times (hereafter referred as RS_Average).
- Entropy-based active learning: We calculate the entropy of each document based on (30), sort documents by their entropy values and feed from documents with top values to those with bottom values as training data. (designated as AL(Entropy))
- Gibbs error based active learning: We calculate the Gibbs error of each document based on (34), sort documents by their Gibbs error values and select the documents with top values as training data. (designated as AL(GibbsError))
- Proposed active learning: We evaluate the method using either unigrams (Unigram) or predicate-argument relations (PAS). The resultant method is referred as AL(Informativity_Unigram) and AL(Informativity_PAS), respectively.
- Conventional committee-based active learning: We evaluate the committee based method based on (22), using the confidence score produced by TEES. We estimate the informativity using either unigrams (Unigram) or predicate-argument relations (PAS) for the proposed statistical method. The resultant method is referred as AL(Conventional Committee_Unigram) and AL(Conventional Committee_PAS), respectively.

**Fig. 2:**
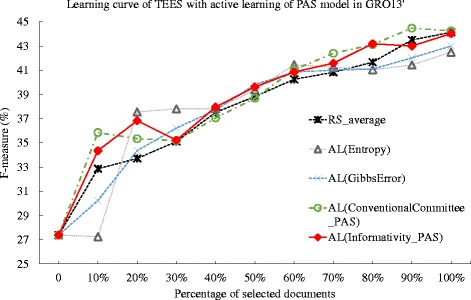
Comparison of active learning with informativity based, entropy-based, Gibbs error based, and conventional committee based method, and random selection against GRO’13 dataset. The learning curves for the TEES system under active learning (AL), using the Gibbs error based method (Gibbs Error), entropy based method(Entropy), conventional committee based method (ConventionalCommittee) and the proposed informativity method (Informativity), as well as the random selection (RS), when tested against the GRO’13 task dataset. The active learning method uses the predicate-argument relation (PAS) model

**Fig. 3:**
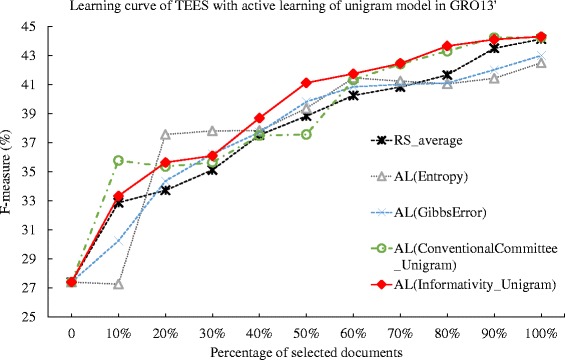
Comparison of active learning with informativity based, entropy-based, Gibbs error based, and conventional committee based method, and random selection against GRO’13 dataset. The learning curves for the TEES system under active learning (AL), using the Gibbs error based method (Gibbs Error), entropy based method(Entropy), conventional committee based method (ConventionalCommittee) and the proposed informativity method (Informativity), as well as the random selection (RS), when tested against the GRO’13 task dataset. The active learning method uses the unigram model

We first apply those methods to the dataset of GRO’13 [19] and measure the performance change of the TEES system with the incremental feed of the training data. We summarize the deficiency for each method in Table 7. The proposed active learning methods and the conventional committee-based methods achieve deficiency value of less than 1, while the entropy and Gibbs error method achieve a deficiency higher than 1, suggesting that the entropy and Gibbs error methods do not perform better than that of random selection. Particularly, the AL(Informativity_Unigram) method achieves the best deficiency of 0.760, while the corresponding conventional committee based method achieves the performance of 0.832 in AL(ConventionalCommittee_Unigram), which is an 8.65 % improvement for the informativity based method over that of conventional committee-based method. However, when using the PAS model, the AL(Informativity_PAS) achieves deficiency of 0.845, which is 1.78 % worse than that of the committee-based method, whose deficiency is 0.830. In addition, when comparing the performance of the methods using the PAS and unigram, we notice that using the unigram, the proposed informativity method shows an 10.1 % improvement over that using PAS model, yet this is not evident in the committee-based method. The results suggest that the proposed informativity method performs best when using the unigram model in the GRO’13 dataset. We then plot the learning curves for each method in Figs. 2 and 3. In Fig. 3, the AL(Informativity_Unigram) method is consistently performing over the other methods after 50 % of the documents are selected, which also explains the results in the comparison of deficiency values. In addition, in the comparison of average number of instances per ontological concept provided in [41], the GRO’13 dataset have 13 instances per concept, while such value for GE’13 dataset is 82. This also suggests that in datasets such as GRO’13 whose document annotation may not be abundant, the active learning method using the unigram may perform better than the PAS model. However, the experiment result in the GRO’13 dataset indicates that the proposed informativity based active learning method with unigram model can show better performance than the conventional committee-based, the entropy based and the Gibbs error based active learning methods.

**Table 7:** Deficiencies of sample selection methods for event extraction against the GRO’13, CG’13 and GE’13 datasets

Method	GRO’13	CG’13	GE’13
RS_Average	1	1	1
AL(Entropy)	1.017	1.226	0.854
AL(GibbsError)	1.039	0.993	0.850
AL(ConventionalCommittee_PAS)	0.830	0.589	0.439
AL(ConventionalCommittee_Unigram)	0.832	0.788	0.263
AL(Informativity_PAS)	0.845	**0.581**	0.872
AL(Informativity_Unigram)	**0.760**	0.768	**0.139**

**Fig. 4:**
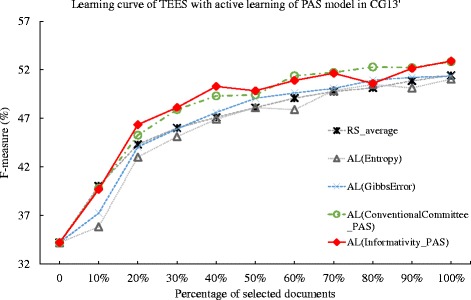
Comparison of active learning with informativity based, entropy-based, Gibbs error based, and conventional committee based method, and random selection against CG’13 dataset. The learning curves for the TEES system under active learning (AL), using the Gibbs error based method (Gibbs Error), entropy based method(Entropy), conventional committee based method (ConventionalCommittee) and the proposed informativity method (Informativity), as well as the random selection (RS), when tested against the CG’13 task dataset. The active learning method uses the predicate-argument relation (PAS) model

**Fig. 5:**
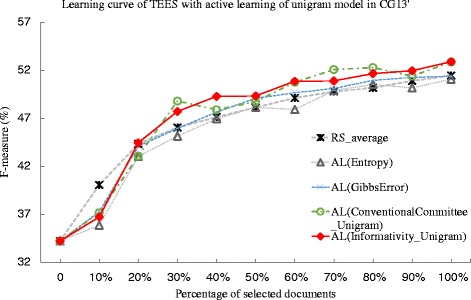
Comparison of active learning with informativity based, entropy-based, Gibbs error based, and conventional committee based method, random selection against CG’13 dataset. The learning curves for the TEES system under active learning (AL), using the Gibbs error based method (Gibbs Error), entropy based method(Entropy), conventional committee based method (ConventionalCommittee) and the proposed informativity method (Informativity), as well as the random selection (RS), when tested against the CG’13 task dataset. The active learning method uses the unigram model

We then carry out a similar experiment using the CG’13 dataset. We summarize the deficiency for each method in the Table 7. In this experiment, the Gibbs error based approach achieves the deficiency value of less than 1, while the deficiency for the entropy based method is 1.226. Comparing the PAS and unigram model, the deficiency values for PAS model are generally better than those of unigram model. For instance, in the committee-based method, the percentage of deficiency difference is 25.3 %. Similarly in the proposed informativity method, there is a 24.3 % change in the deficiency value. This may suggest that the PAS model may be more suitable for the CG’13 dataset. In addition, while comparing the proposed informativity method and committee-based method, the informativity method achieves better deficiency value over the committee-based method. In terms of deficiency difference, the improvements are 0.020 and 0.008, for PAS and unigram feature, respectively, which is a less obvious improvement for the informativity method. However, this also suggest that the PAS feature may be more sensitive than that of unigram in the CG’13 dataset. Note that one of the specialties in CG’13 dataset is that only a single relation type of *Equiv* is defined. *Equiv* is a symmetric and transitive binary relation to identify entity mentions as being equivalent in the sense of referring to the same real-world entity [42]. Such relation is not evaluated in the GRO’13 or GE’13 dataset. The better performance of PAS model over unigram model may due to that the PAS model is more stable for identification of equivalent entity mentions than the unigram model. The learning curves for the active learning method are plotted in Figs. 4 and 5.

**Fig. 6:**
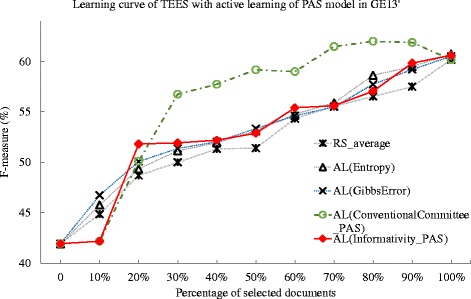
Comparison of active learning with informativity based, entropy-based, Gibbs error based, and conventional committee based method, and random selection against GE’13 dataset. The learning curves for the TEES system under active learning (AL), using the Gibbs error based method (Gibbs Error), entropy based method(Entropy), conventional committee based method (ConventionalCommittee) and the proposed informativity method (Informativity), as well as the random selection (RS), when tested against the GE’13 task dataset. The active learning method uses the predicate-argument relation (PAS) model

**Fig. 7:**
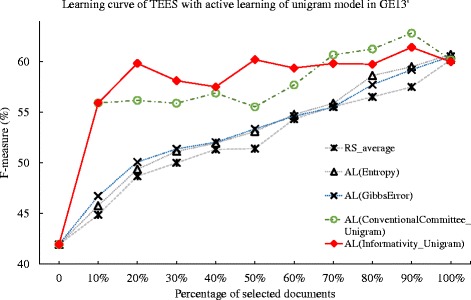
Comparison of active learning with informativity based, entropy-based, Gibbs error based, and conventional committee based method, and random selection against GE’13 dataset. The learning curves for the TEES system under active learning (AL), using the Gibbs error based method (Gibbs Error), entropy based method(Entropy), conventional committee based method (ConventionalCommittee) and the proposed informativity method (Informativity), as well as the random selection (RS), when tested against the GE’13 task dataset. The active learning method uses the unigram model

We extend the aforementioned active learning methods to the GE’13 dataset, and the Table 7 summarize the deficiency of the methods. In Table 7, all methods achieve deficiency values less than the random selection. The method of Gibbs error based approach achieve the deficiency of 0.850, while the deficiency for the entropy method is 0.854. The proposed active learning methods using the unigram shows a more obvious improvement than that using PAS. For instance, in the committee-based method, there is an improvement of 40.1 % for the unigram model over the PAS model. This may suggest that, against the GE’13 dataset, the unigram feature is more suitable for proposed method than that of the PAS feature. We notice a more obvious improvement for the unigram model in the informativity method. Particularly, the best performing AL(Informativity_Unigram) achieve a deficiency value of 0.139. While the corresponding committee-based method achieve the deficiency of 0.263 in AL(ConventionalCommittee_Unigram). We plot the learning curves in Figs. 6 and 7. In the Fig. 7, the active learning method using unigram generally shows obvious improvement over the baseline of random selection method, yet the active learning method using PAS show less significant improvement over the baseline method. This may due to the fact that the ontology defined in GE’13 task is generally less complicated than that in GRO’13 and CG’13. In addition, the document annotation in the GE’13 dataset may be abundant, as the average number of instances per ontological concept in GE’13 dataset is 82, above six times more than that of GRO’13 dataset [41]. Given the dataset with less complicated ontological concepts and abundant training data of document annotation, the unigram model may show obvious improvement for active learning methods.

#### Active learning methods using additional features

**Fig. 8:**
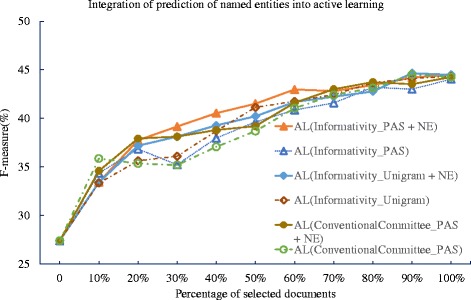
Integration of named entity recognition into active learning with PAS and n-grams against GRO’13 dataset. The learning curves for the TEES system under the proposed informativity method using predicate-argument relation (PAS) and unigram model, as well as the conventional committee (ConventionalCommittee) based active learning method as the benchmark. In contrast, each method is integrated with the output from the named entity recognition result (NE)

**Incorporation of event participants** We evaluate the active learning method that is incorporated with the recognition of gene/protein names for event extraction, as illustrated in Formula (14). We show the performance of the TEES system, with active learning method that is either with or without using the gene/protein names. Such experiment is carried out using the GRO’13 dataset. The experiment results are plotted in Fig. 8 and we summarize the deficiency values in the Table 8. In the Table 8, the incorporation of gene/protein names shows positive effects towards the active learning method for event extraction, for both of bag of n-gram or PAS method. By using the gene/protein names, the deficiency for the active learning method using PAS is further improved from 0.845 to 0.589, which is a 30.3 % improvement. Yet in the unigram model of the informativity method, the improvement is rather less significant of 7.1 %, which may suggest that some named entities are already captured as n-grams, thus redundant.

**Table 8:** Deficiencies of active learning methods with and without integrating the prediction of named entities (NE) against GRO’13 dataset

Method	GRO’13
RS_Average	1
AL(ConventionalCommittee_PAS)	0.830
AL(ConventionalCommittee_PAS + NE)	0.693
AL(Informativity_PAS)	0.845
AL(Informativity_PAS + NE)	**0.589**
AL(Informativity_Unigram)	0.760
AL(Informativity_Unigram + NE)	0.706

In addition, we notice similar improvement of the conventional committee-based method by incorporating the information of event participants into the part of statistical informativity estimation, from 0.830 (i.e. ConventionalCommittee_PAS) to 0.693 (i.e. ConventionalCommittee_PAS + NE), a 16.5 % improvement. However, this improvement is significantly less than that for our proposed method, which may indicate that the confidence scores of the TEES used by the conventional committee-based method hamper the effects of event participants.

**Fig. 9:**
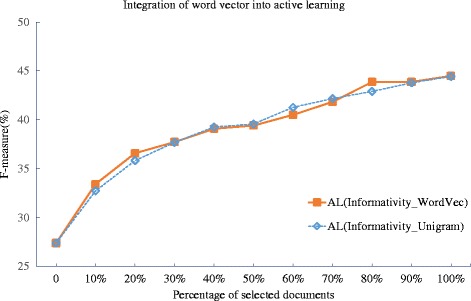
Evaluation of incorporation of the word vector method into active learning with n-grams against GRO’13 dataset. The word vector is applied into the active learning method to solve the out-of-vocabulary (OOV) issue that exists in the unigram model. For the unknown unigram, its score is replaced by the top-25 most similar known unigrams

**Dealing with OOV issue with word similarity** The n-gram model is based on the ‘registered’ n-grams that occur in the training data, which has the issue of Out-of-Vocabulary (OOV) words. We solve this by using the word2vec toolkit to find top-*k* words that are closest to a given OOV word in the test data and to use their weights to estimate the weight of the OOV word. The results of evaluating the word vector incorporation against the GRO’13 dataset are plotted in Fig. 9, and the deficiency is summarized in Table 9. Note that the experiments about OOV word handling are carried out only for events, excluding relations, observing that the relations of the BioNLP-ST’13 tasks are little affected by the OOV issue, since they are not associated with trigger words. By using the word similarity, the n-gram model method is further improved, as the deficiency of n-gram model goes from 0.790 to 0.769, an improvement of 2.66 %. The rather less significant improvement may suggest that such OOV issue is rather not prevalent in the GRO’13 dataset.

**Table 9:** Deficiencies of using word vector to solve the Out-Of-Vocabulary(OOV) issue for the unigram model

Method	GRO’13
RS_Average	1
AL(Informativity_Unigram)	0.790
AL(Informativity_WordVec)	**0.769**

### Linear combination of n-gram and predicate-structure relation features

Lastly, we linearly combine the proposed n-gram and predicate-structure relation features for the active learning, as expressed in Eq. (17), and to understand which of the active learning methods proposed in this paper are more important towards the overall performance.

We use four weight combinations of (*α*=0.8, *β*=0.1, *γ*=0.1), (*α*=0.1, *β*=0.8, *γ*=0.1), and (*α*=0.1, *β*=0.1, *γ*=0.8), as well as the equal distribution of weight (*α*=0.33, *β*=0.33, *γ*=0.33). The method of AL(Informativity_PAS + NE) is used as the benchmark, as it is the best performing method in the previous experiments in the GRO’13 dataset. Note that the AL(Informativity_PAS + NE) corresponds to the weight combination of (*α*=0, *β*=1, *γ*=1). Additionally, we also use the benchmark of only using the named entity for the active learning, i.e the weight combination of (*α*=0, *β*=0, *γ*=1), to check if simply using the total number of recognized named entities be sufficient for the active learning method.

**Fig. 10:**
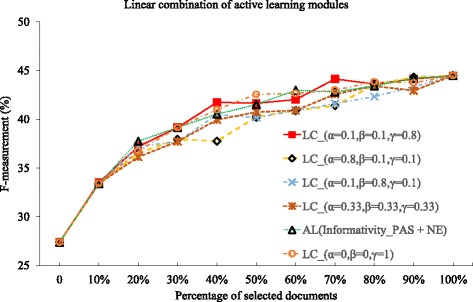
Evaluation of linear combination of active learning methods against GRO’13 dataset. The active learning modules are assigned with different weights and combined linearly. Different weight assignment strategies are compared

The results of comparison are plotted in Fig. 10, and we summarize the deficiency values in Table 10. Overall, the weight combination of (*α*=0.1, *β*=0.1, *γ*=0.8) shows the best performance (deficiency 0.563). Compared to PAS or unigram-based statistics, the incorporation of event participants has the most effect on the best performance. Note, however, that the model of using only the event participants, i.e., the weight combination of (*α*=0, *β*=0, *γ*=1), achieves the deficiency of 0.583, higher than the best deficiency, which indicates that the PAS or n-gram based statistics are complementary to event participants.

**Table 10:** Deficiencies of linear combination of active learning methods

Method	GRO’13
RS_Average	1
AL(Informativity_PAS + NE)	0.589
LC_(*α*=0.33, *β*=0.33, *γ*=0.33)	0.740
LC_(*α*=0.8, *β*=0.1, *γ*=0.1)	0.772
LC_(*α*=0.1, *β*=0.8, *γ*=0.1)	0.752
LC_(*α*=0.1, *β*=0.1, *γ*=0.8)	**0.563**
LC_(*α*=0, *β*=0, *γ*=1)	0.583

### Evaluation of active learning method for NER task

We apply the active learning method into NER task as expressed in Eq. (18), and follow the similar experiment design. Each sample selection method starts with the same held-out labeled development dataset for model initialization and a pool of unlabeled training dataset for selection. In each round, 10 % of the unlabeled documents in the training dataset are selected by different sample selection strategies. For evaluation, we report the performance of NER system trained with the selected training document in each round, against the same held-out test dataset following the official evaluation procedure.

The sample selection strategies are as follows:

- Random selection: We randomly split the training dataset into 10 bins in advance, one bin is randomly chosen in each round. Following 10-fold cross validation, we report the averaged performance in each round. (hereafter referred to as RS_Average)
- Entropy-based active learning: The entropy of documents are calculated, and select documents by their entropy values, from the top to bottom. (designated as AL(Entropy))
- Maximum Gibbs Error based active learning: Similar to the entropy-based method, but uses the Gibbs error, as introduced in [33]. (designated as AL(GibbsError))
- Proposed active learning method using informativity scoring only: Use the aforementioned system in Eq. (18), and selects documents based on their informativity scores. (designated as AL(Informativity))
- Conventional committee-based active learning: We evaluate the committee based method based on (22), using the confidence score produced by NER system. The resultant method is referred as AL(ConventionalCommittee).

**Fig. 11:**
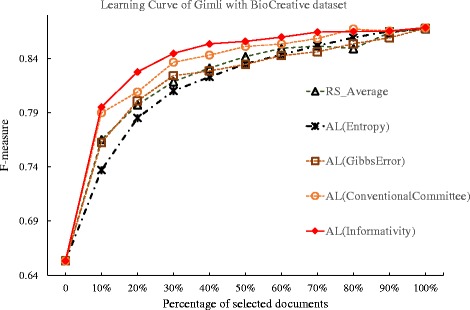
Comparison of active learning with informativity based, entropy-based, Gibbs error based, and conventional committee based method, and random selection against BioCreative dataset. The learning curves for the Gimli system under active learning (AL), using the Gibbs error based method (Gibbs Error), entropy based method(Entropy), conventional committee based method (ConventionalCommittee) and the proposed informativity method (Informativity), as well as the random selection (RS), when tested against the BioCreative task dataset

We applied these methods to the BioCreative dataset and plotted the learning curve of Gimli in Fig. 11, and summarized their deficiency values in Table 11. In Fig. 11, the proposed active learning method show steady improvement over the other methods in most rounds. Based on the deficiency comparison in Table 11, the proposed method achieved a deficiency value of 0.514, while the deficiency for the conventional committee based method is 0.684.

**Table 11:** Deficiencies of sample selection methods against the BioCreative and CoNLL datasets

Method	BioCreative	CoNLL
RS_Average	1	1
AL(Entropy)	1.171	0.737
AL(GibbsError)	1.045	0.885
AL(ConventionalCommittee)	0.684	0.763
AL(Informativity)	**0.514**	**0.575**

**Fig. 12:**
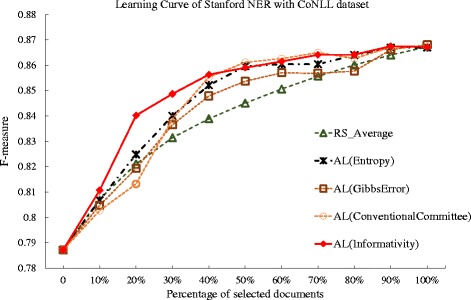
Comparison of active learning with informativity based, entropy-based, Gibbs error based, and conventional committee based method, and random selection against CoNLL dataset. The learning curves for the Gimli system under active learning (AL), using the Gibbs error based method (Gibbs Error), entropy based method(Entropy), conventional committee based method (ConventionalCommittee) and the proposed informativity method (Informativity), as well as the random selection (RS), when tested against the CoNLL task dataset

We carried out similar experiments with the CoNLL dataset, and the learning curves are plotted in Fig. 12, and the deficiencies are compared in Table 11. In Fig. 12, the proposed active learning method outperforms the other methods; and in terms of deficiency, the proposed method achieves 0.575 in the deficiency, a nearly 42 % improvement over the random selection. In contrast, the benchmark of Entropy and Gibbs error based approaches also are shows deficiency value of less than 1, yet their improvement over the random selection is nearly 26 % and 11 %. The deficiency for the conventional committee based method is 0.763. The experiment results in the BioCreative and CoNLL datasets indicate that the proposed informativity based method can show better performance than the conventional committee-based method, as well as the Entropy and Gibbs error based methods.

## Conclusions

In this study, we proposed a novel active learning method for ontological event extraction, which is more complex than the simple PPI extraction. Our method measures the collective ‘informativity’ for unlabeled documents, in terms of the potential likelihood of biological events unrecognizable for the event extraction system. We evaluated the proposed method against the BioNLP Shared Tasks datasets, and showed that our method can achieve better performance than other previous methods, including entropy and Gibbs error based methods and the conventional committee-based method. In addition, the incorporation of named entity recognition into the active learning for event extraction and the unknown word handling further improved the active learning method. Finally, we adapted the active learning method into named entity recognition tasks and showed that the method also improved the document selection for manual annotation of named entities.

## References

[1] Hirschman L, Yeh A, Blaschke C, Valencia A (2005). Overview of BioCreAtIvE: critical assessment of information extraction for biology. BMC Bioinformatics.

[2] Šarić J, Jensen LJ, Ouzounova R, Rojas I, Bork P (2006). Extraction of regulatory gene/protein networks from Medline. Bioinformatics.

[3] Kim JD, Nguyen N, Wang Y, Tsujii J, Takagi T, Yonezawa A (2012). The Genia Event and Protein Coreference tasks of the BioNLP Shared Task 2011. BMC Bioinformatics.

[4] Settles B (2012). Active learning. Synth Lect Artif Intell Mach Learn.

[5] Chen Y, Cao H, Mei Q, Zheng K, Xu H (2013). Applying active learning to supervised word sense disambiguation in MEDLINE. J Am Med Inform Assoc.

[6] Tomanek K, Hahn U (2009). Reducing Class Imbalance During Active Learning for Named Entity Annotation. Proceedings of the Fifth International Conference on Knowledge Capture.

[7] Tomanek K, Hahn U. Semi-Supervised Active Learning for Sequence Labeling. In: Proceedings of the Joint Conference of the 47th Annual Meeting of the ACL and the 4th International Joint Conference on Natural Language Processing of the AFNLP: 2009. p. 1039–47.

[8] Tomanek K, Hahn U. A Comparison of Models for Cost-Sensitive Active Learning. In: International Conference on Computational Linguistics (Coling) 2010: Posters: 2010. p. 1247–1255.

[9] Zhang J, Yuan H. A Certainty-Based Active Learning Framework of Meeting Speech Summarization. In: Computer Engineering and Networking: 2014. p. 235–42.

[10] Lewis DD, Catlett J. Heterogeneous Uncertainty Sampling for Supervised Learning. In: Proceedings of the Eleventh International Conference on Machine Learning: 1994. p. 148–56.

[11] Seung HS, Opper M, Sompolinsky H (1992). Query by committee. Proceedings of the Fifth Annual Workshop on Computational Learning Theory.

[12] Fu Y, Zhu X, Li B (2013). A survey on instance selection for active learning. Knowl Inf Syst.

[13] Schütze H, Velipasaoglu E, Pedersen JO (2006). Performance Thresholding in Practical Text Classification. Proceedings of the 15th ACM International Conference on Information and Knowledge Management. CIKM ’06.

[14] Tomanek K, Laws F, Hahn U, Schütze H (2009). On Proper Unit Selection in Active Learning: Co-Selection Effects for Named Entity Recognition. Proceedings of the NAACL HLT 2009 Workshop on Active Learning for Natural Language Processing.

[15] Wallace BC, Small K, Brodley CE, Trikalinos TA (2010). Active Learning for Biomedical Citation Screening. Proceedings of the 16th ACM SIGKDD International Conference on Knowledge Discovery and Data Mining. KDD ’10.

[16] Cui B, Lin H, Yang Z (2009). Uncertainty sampling-based active learning for protein-protein interaction extraction from biomedical literature. Expert Syst Appl.

[17] Zhang HT, Huang ML, Zhu XY (2012). A unified active learning framework for biomedical relation extraction. J Comput Sci Technol.

[18] Kim JD, Ohta T, Pyysalo S, Kano Y, Tsujii J (2009). Overview of BioNLP’09 Shared Task on Event Extraction. Proceedings of the BioNLP 2009 Workshop Companion Volume for Shared Task.

[19] Kim JJ, Han X, Lee V, Rebholz-Schuhmann D (2013). GRO Task: Populating the Gene Regulation Ontology with events and relations. Proceedings of the BioNLP Shared Task 2013 Workshop.

[20] Nédellec C, Bossy R, Kim JD, Kim JJ, Ohta T, Pyysalo S, Zweigenbaum P (2013). Overview of BioNLP Shared Task 2013. Proceedings of the BioNLP Shared Task 2013 Workshop.

[21] Björne J, Heimonen J, Ginter F, Airola A, Pahikkala T, Salakoski T (2011). Extracting Complex Biological Events with Rich Graph-Based Feature Sets. Comput Intell.

[22] McCallum A, Nigam K (1998). Employing EM and Pool-Based Active Learning for Text Classification. Proceedings of the Fifteenth International Conference on Machine Learning. ICML ’98.

[23] Culotta A, McCallum A (2005). Reducing Labeling Effort for Structured Prediction Tasks. Proceedings of the 20th National Conference on Artificial Intelligence - Volume 2..

[24] Settles B, Craven M (2008). An Analysis of Active Learning Strategies for Sequence Labeling Tasks. Proceedings of the Conference on Empirical Methods in Natural Language Processing. EMNLP ’08.

[25] Björne J, Ginter F, Salakoski T (2012). University of Turku in the BioNLP’11 shared task. BMC Bioinformatics.

[26] Sagae K, Miyao Y, Tsujii J (2007). HPSG Parsing with Shallow Dependency Constraints. Proceedings of the 45th Annual Meeting of the Association of Computational Linguistics.

[27] Corder GW, Foreman DI (2009). Nonparametric Statistics for Non-statisticians: a Step-by-step Approach.

[28] Campos D, Matos S, Oliveira JL (2013). Gimli: open source and high-performance biomedical name recognition. BMC Bioinformatics.

[29] Mikolov T, Sutskever I, Chen K, Corrado GS, Dean J (2013). Distributed Representations of Words and Phrases and their Compositionality. Advances in Neural Information Processing Systems 26.

[30] Schapire RE, Freund Y, Bartlett P, Lee WS (1998). Boosting the margin: A new explanation for the effectiveness of voting methods. Ann Stat..

[31] Vlachos A. Active learning with support vector machines: School of Informatics University of Edinburgh; 2004, pp. 12–14.

[32] Shannon CE (2001). A mathematical theory of communication. ACM SIGMOBILE Mobile Comput Commun Rev.

[33] Cuong NV, Lee WS, Ye N, Chai KMA, Chieu HL (2013). Active Learning for Probabilistic Hypotheses Using the Maximum Gibbs Error Criterion. Advances in Neural Information Processing Systems 26.

[34] Pyysalo S, Ohta T, Ananiadou S (2013). Overview of the Cancer Genetics (CG) task of BioNLP Shared Task 2013. Proceedings of the BioNLP Shared Task 2013 Workshop.

[35] Kim JD, Wang Y, Yasunori Y (2013). The Genia Event Extraction Shared Task, 2013 Edition - Overview. Proceedings of the BioNLP Shared Task 2013 Workshop.

[36] Finkel JR, Grenager T, Manning C (2005). Incorporating Non-local Information into Information Extraction Systems by Gibbs Sampling. Proceedings of the 43rd Annual Meeting on Association for Computational Linguistics. ACL ’05.

[37] Tjong Kim Sang EF, De Meulder F (2003). Introduction to the CoNLL-2003 Shared Task: Language-Independent Named Entity Recognition. Proceedings of CoNLL-2003.

[38] Smith L, Tanabe L, Ando R, Kuo CJ, Chung IF, Hsu CN, Lin YS, Klinger R, Friedrich C, Ganchev K, Torii M, Liu H, Haddow B, Struble C, Povinelli R, Vlachos A, Baumgartner W, Hunter L, Carpenter B, Tsai R, Dai HJ, Liu F, Chen Y, Sun C, Katrenko S, Adriaans P, Blaschke C, Torres R, Neves M, Nakov P, Divoli A, Mana-Lopez M, Mata J, Wilbur WJ (2008). Overview of BioCreative II gene mention recognition. Genome Biol.

[39] Zhu J, Wang H, Yao T, Tsou BK. Active Learning with Sampling by Uncertainty and Density for Word Sense Disambiguation and Text Classification. In: Proceedings of the 22nd International Conference on Computational Linguistics (Manchester, UK: Coling 2008 Organizing Committee): 2008. p. 1137–1144.

[40] Baram Y, El-Yaniv R, Luz K (2004). Online choice of active learning algorithms. J Mach Learn Res.

[41] Kim JD, Kim J-j, Han X, Rebholz-Schuhmann D (2015). Extending the evaluation of genia event task toward knowledge base construction and comparison to gene regulation ontology task. BMC Bioinformatics.

[42] Pyysalo S, Ohta T, Rak R, Rowley A, Chun HW, Jung SJ, Choi SP, Tsujii J, Ananiadou S (2015). Overview of the cancer genetics and pathway curation tasks of bionlp shared task 2013. BMC Bioinformatics.

